# Management and Best Means of Preserving the Deciduous Teeth

**Published:** 1868-04

**Authors:** W. E. Magill


### MANAGEMENT AND BEST MEANS OF PRESERVING THE DECIDUOUS TEETH.

BY W. E. MAGILL.

Read before the Lake Erie Dental Association, February 4, 1868.

GENTLEMEN: The discussion which I am called upon to open by the presentation of this paper, is to be confined to the "Management and best Means of Preserving Deciduous Teeth;" and let me express the hope that in placing this subject first upon the list for discussion, we have correctly indicated our appreciation of its importance.

Permit me, also, here and now, to frankly own my inability to do this subject justice. It is my *duty* to open this discussion. It shall be my *aim* to block out some of our professional work; to indicate the direction in which one branch or class of professional duties may lead us; but not to play tutor, or claim ability to act as pilot in the exploration of such vast scientific regions as might be properly included in a free discussion of this interesting subject.

The management of children's teeth properly will be considered under two heads or principal divisions of treatment. First, hygienic or prophylactic, having in view *prevention.* Second, remedial or redemptive, having in view *cure.*

The first and highest object of treatment is to secure the full and proper development of the teeth of the child—a set of perfect teeth in a perfect body. Each one of us has opportunity to observe the sad results of sickness and consequent arrest of development, as plainly marked on the little sufferer's teeth, as are changes in the earth's surface shown by her geologic formations. If, then, the teeth of children are subject to laws of health and disease which govern the general system, is it not of the first importance to us, as professional men, and to our patients who seek advice, that we become intimately acquainted with this general system, understand what it should be in health, be quick to detect deviations from that condition, and prompt in the application of remedies?

I cannot forego this opportunity to protest against all quackery in our profession — all assumption of unacquired wisdom — all attempts on our part to impose upon a confiding public by therapeutic experiments. Let us inform ourselves — let us be fit and competent to diagnose disease, and familiar with proper remedies before we expect success in this inviting field.

In my own mind there is no doubt that the proper *time* to commence treatment is early in the period of gestation, by furnishing the *mother* with such abundant nourishment of teeth-forming material that not only shall the embryo child have all its needs in that direction supplied, but her own teeth shall be protected against the softening process so common at that period. As early as the third month of fœtal life the tooth germs develope, and he who undertakes to supply the coming want for tooth ingredients must be prepared to fill orders.

Our books tell us that teeth are composed of

| | Temporary. | Adult. | Adult. |
|---|---|---|---|
| Organic matter | 31.55 | 28.20 | 26.81 |
| Phosphate of Lime | 61.23 | 60.16 | 66.42 |
| " " Magnesia | | 1.66 | 0.62 |
| Carbonate of Lime | 4.71 | 7.04 | 5.63 |
| Chloride of Sodium / " " Potasium | 2.51 | 2.66 | 0.10 |
| | 100.00 | 99.72 | 99.58 |

The temporary teeth being intended only to remain for a short period, do not require such density of structure as those provided for use during adult life, and the low degree of density of the temporary set many explain their proneness to decay.

Phosphate of Lime, then, being largely in the majority among elements for building tooth structures, it becomes important to know whence a supply may be derived. Unquestionably the best *form* in which to exhibit phosphates is as prepared by Nature, in the breads and meats selected for use. They may be administered as medicine, directly, but I would incline to the former mode, as establishing better habits of diet and likely to continue the desired effect for longer periods, aside from any discussion as to the *immediate* benefits.

M. Collas, known in regard to his researches upon the phosphates, is quoted thus: "In pregnant women or wet nurses, there is an absence of phosphate of lime in the organism. The phosphate passes in a great part into the blood and milk, so as to serve for the consolidation of the bones of the infant, which are at birth in a cartilagenous state."

He advises the following preparations: 1st. Solution of phosphate of soda. Eight grammes of phosphate in spring water. Take two or three glasses per day.

2d. *Phosphoric Lemonade.*—Phosporic Acid two grammes, in a litre of spring water, taken occasionally as a beverage.

*Hydrated Phosphate of Lime Milk.*—Ordinary hydrated phosphate of lime fifty grammes, water one hundred grammes, mixed in a mortar, and passed through a strainer or fine sieve. One, two, or three spoonfuls to be taken, especially in soup. This is the best way of administering phosphate of lime to rickety children."

But after birth tooth-development proceeds, and for two years or more the same importance attaches to the food of the infant, and of course of the mother, unless the child be fed by hand. In this latter case my own experience is in favor of Liebig's formula:

Liebig's Formula.—Half an ounce of wheaten flour, and an equal quantity of malt flour, 7¼ grains bicarbonate of potassa, and one ounce of water are to be well mixed. Five ounces of cow's milk are then added, and the whole put over a gentle fire. When the mixture begins to thicken it is removed from the fire, stirred during five minutes, again heated and stirred until it becomes quite fluid, and finally is made to boil. Then strain through a cloth or fine sieve. No sugar is needed.

To be guarded, however, in this way: whenever excessive looseness of the bowels comes on, the 7½ grains potash shall give place to 20 grains prepared chalk, and if the chalk brings on constipation, both that and the potash may be dispensed with, and sometimes better results obtained than by the use of either. It is best to be careful that good milk, from one cow, is procured.

The importance of proper food at this period of life is very strikingly shown by the experiment of a London surgeon who exhibited before the Pathological Society of that city the bones of a dog, in which, as a puppy, rickets had been artificially induced by early removal from its mother, and feeding it on bread and meat, with only milk enough to keep it from starving altogether. His theory is that, by the improper dieting of children, an excess of phosphoric acid is formed, which carries off with it an excess of lime, and thus softens the bones. He questioned if rickets was ever congenital.

Nearly twenty years since Mr. Nasmyth gave the following opinion:

"Pursuing the deductions obtained from a consideration of the specific gravity, particularly that exhibited by the decayed portion of human ivory, conjoined with the proportion of its constituents, we may infer that the greater the density of teeth the less likely are they to decay. That the character of the structure of teeth is regulated by the climate, food, and habits of individuals may, I think, be fairly deduced from the circumstance of such variety of density being displayed by the teeth of the single family of the elephant, spread over a great extent of the surface of the globe. The practical inference fairly deducible from the latter observation is that attention to proper regimen and to the general treatment and health of mankind, will contribute directly in favor of perfect and healthy development of their teeth. That circumstances of this kind do act directly in regulating the development of the teeth of man we see demonstrated by the difference of specific gravity found betwixt those of the native

Indian, (Hindoo) having entirely a vegetable food, and of the European, living on a mixed animal and vegetable diet; the former having a less density than the latter, seems to warrant the inference that animal food is more favorable to the perfect development of the earthy structures. It is remarked, also, by veterinary surgeons, that the development of the teeth of horses is somewhat affected by their food; when fed on hard food their teeth being more easily developed than on soft.

The exploration in regard to New York "*swill milk*" had not then been made, or Mr. Nasmyth might have had another illustration of the truth of his theory.

In a discussion before the Brooklyn Association, Dr. Latimer said: "With a view of demonstrating the great difference in various teeth, I selected two corresponding teeth of the same age, both equally dried and of equal weight, then by burning out the organic elements I found a loss of four grains more in one tooth than in the other. A second experiment showed a loss of four and one-half grains more in the chalky tooth than in one better calcified. For the third test I selected two teeth, each weighing one dwt, equally dried, but as different as possible in density. After burning them I found that one had lost nine grains in weight — the other only three." Other experiments showed a loss in the organic constituents of from three to ten grains per tooth.

Members of our profession are not without experience in the direction of constitutional treatment for perfecting tooth development, and very gratifying success has attended such effort. Enough to urge us all to careful study and thorough preparation for labor in this wide field of useful professional distinction opening before us.

We must not, however, lose sight of the fact that *adverse* influences exist in many constitutions — that defects are transmitted — that taints come down through long lines of descent, and that by such considerations sometimes our successes or our failures are to be measured.

It is also an interesting fact which each one of us has probably observed that, in the same family, subject, as nearly as may be to the same rules of diet, and all in fine health, some members will have excellent teeth and others very defective teeth. Generally, too, we find that the *good* teeth are in the *boy's* mouths. Indeed, it is a sad fact that not to *vanity*, as some insinuate, but to dire necessity, are due the frequent demands of young *ladies* upon our skill.

It seems probable that the out-door sports of boys, furnishing them more fresh air, and their active exercise giving them an appetite for meat and other hearty food, may largely account for the difference; but there are subtleties in constitutional tendencies and assimilative power which I do not pretend to fathom.

But having, by proper food, secured the development of sound teeth, is our mission accomplished? By no means. The conjectural theory that life mysteriously endows living matter with a defensive virtue which enables it to resist the chemical and other powers acting regularly upon inorganic or dead matter is no longer tenable. "Life cannot make the brute materials which it uses live longer than that which it leaves unused; but it has the power of making them anew and building them up into a certain shape for the time they are made to last. In short, life rests on the metamorphosis or renewal of the body; as this renewal is more thorough, the individual is more perfect and fulfills better and more completely the duties of its position. If it stops altogether, the body is no longer living. If it partially stops, the order of normal phenomena is disarranged, and ease is expelled—there is a state which we call *dis*-order or *dis*-ease."

Briefly, now, let us thus sum up our conclusions: It is within our province and within the bounds of possibilities, to *cultivate* or assist in the production of good teeth.

If teeth are more dense in structure, and contain larger proportions of the inorganic elements, they are, therefore, better protected against the ordinary causes of decay.

If we would retain the teeth in perfect health, we must sustain the healthy tone of the general system, and furnish materials for that renewal or reproduction which is the essence of life.

But secondly: These beautiful structures sometimes break — these cunning tissues decay — what then?

If the general condition of the mouth seems good, and decay confined to the grinding surface of molars or approximal surfaces of bicuspids, I would expect to meet the demands of the occasion by filling with gold, or, more frequently, with amalgam. Often the proximal cavities have broken walls, and are so shallow that to cut grooves would be too painful, or risk the exposure of the pulp, which, in these temporary teeth, approaches the surface so closely that one may come upon it unexpectedly. In these cases I fill with "osteoplastic." Whatever may be the filling, let us always make the operation *gentle*, and, if possible, pleasant to the little patient. To the parent let us say: These fillings may need renewal soon, but even if within a few weeks or months, they will have done good service in relieving sensitiveness of tooth structure, and familiarizing the child with Dental operations. Children carefully treated will often ask to come again, as a privilege; and after their confidence is secured, and a proper sympathy established, it is surprising what heroism the little soldiers will show. Then, when the six-year molars appear, your subject is ready to have a first-class filling inserted, if need be.

But sometimes pulps are exposed, past all hope of capping or protecting with "osteoplastic," and parent or child, or both, demand relief by extraction of the offending member. My own rule is to put off as long as possible, or as near to the time of development of permanent teeth as can be done with comfort, the extraction of deciduous teeth. Therefore applications of creosote are made — parents are directed to keep laudanum or camphor, or chloroform for direct application, or to bring the child just as often as they choose to the office. Frequent applications of creosote or a saturated solution of tannin will slowly devitalize the exposed pulp; and as, if left exposed, the same result will be arrived at by inflammation, accompanied by pain, and to be followed by abscess and suppuration. I choose the less painful course, and often remove the pulp and subsequently fill with metal. Abscess, sooner or later, may result, but the alveolar arch is retained in full size, and the permanent teeth do develope well beneath those hollow, dead deciduous teeth.

I know that one who holds an honorable position among the wise men of our profession, in a discussion upon this subject, about a year since, said "there is scarcely a single condition under which the vascular loop or pulp of a deciduous tooth can be safely devitalized, as a remedial measure;" but the same gentleman, during the same discussion, made the broad assertion, "clean teeth are sound teeth, the world over," and I am willing to let one opinion offset the other. I presume, however, the Doctor had in view the treatment with *arsenic*, and that when the question was, whether to promptly extract a deciduous tooth three years before its proper period, or to allay pain, and slowly but surely devitalize the pulp, he would choose the latter course, as the least of two evils. To attempt capping or filling with "osteoplastic," is to inflict needless pain, for if any structure is more sensitive to external influences than the pulp of a permanent tooth, it is the pulp of one of these deciduous teeth.

If decay is of a somewhat general character, affecting the buccal and labial surfaces, I endeavor to secure proper diet, to have the teeth brushed often with tepid water, and a free use of prepared chalk, but not frequently as a *dentifrice*. Sometimes have a wash of lime water used, and always insist on cleanliness.

Our treatment of decay will, of course, depend upon the theory we adopt in regard to its cause or causes. Some persons consider it exclusively a vital process — others exclusively a chemical one.

M. Robin, a French investigator, says: "Caries of teeth is ordinarily due to the softening of the dentine, which becomes fatty, and loses its mineral before its organic elements.

In a discussion before the College of Dentists, London, M. Perkins considered the imperfect formation of the enamel a frequent cause of decay, by food collecting in the interstices and decomposing; and that without chemical action teeth would not decay at all, however predisposed they might be.

Mr. Hulme observed that the causes of caries may be divided into two kinds — the exciting and the predisposing. It was still a question, not fully decided, whether the dentine does not lose its vitality, or have it impaired, before it is attacked by caries. It was quite certain that after severe attacks of illness, teeth which were previously healthy, often passed into a state of rapid decay, the tooth disease not having been produced by the use of medicines, but apparently arising from constitutional causes. The coloring of teeth in jaundice, the reddening of the teeth, and the changes of color which may be noticed from day to day in them, vary with the state of health, and seem to prove that internal changes are constantly taking place quite independent of external agents.

In his chapter on the chemical composition of teeth, Mr. Nasmyth says: "We must bear in mind that the tooth is composed of organized animal and earthy matter. In the exclusively vital process the result would be a derangement in the constitution of the earthy salts, so as to allow them to be decomposed and taken up by the system or the saliva in its ordinary state. The exclusively chemical process, or that capable of being produced by the putrefaction of the food collected about the teeth, would necessarily act on the animal matter, and produce its destruction. Whichever of these processes occurs, a certain degree of faultiness, as it appears to me, must exist in the tooth to admit of the particular part being affected." "In regard to the process of decay, it appears to me that in most cases it is a combination of vital and chemical processes. It cannot be considered exclusively under the influence of either, but at the same time I consider it more the effect of vital than of chemical action. Without any deficiency in the original organization of the tooth, I consider it quite possible for a faulty vitality to originate decay; but the simple presence of food, without any natural deficiency in the tooth, I consider incapable of producing that effect. If, however, there be also the presence of a foreign body, such as may be used for false teeth, then I know it to be possible; and unless great care and cleanliness be practiced, that effect is sure to follow." "The materials of the body are in a constant condition of progression or replacement, by one process or another, and a supply of new material is constantly going on. When these processes are balanced, the functions of the part are perfect; but if there be a subtraction of material without any adequate supply, decay of the part must succeed. We have in the tissue of ivory a cellular structure, furnished with organized animal matter, in the presence of the rows of persistant nuclei, surrounded by the parcels of earthy matter which form the characteristic of the structure. We have, therefore, analagous constituents to other tissues, where the processes of replacement or absorption of old, and nourishment of new parts goes on; we cannot then, deny this tissue the exercise of similar functions. Decay will be developed in the different portions of the tooth in an inverse ratio to the power of resistance. The more perfectly the structure is organized, the greater will be the power of resistance."

Shall we not conclude, then, that in seeking the best means of preserving the deciduous teeth, there is *no* period when we can ignore the benefits and importance of constitutional treatment. That topical applications, whether by filling or otherwise, are mere adjuncts or temporary appliances, awaiting the grander results to be secured through the vital functions.

